# Nuclear overexpression of DNA damage-inducible transcript 4 (DDIT4) is associated with aggressive tumor behavior in patients with pancreatic tumors

**DOI:** 10.1038/s41598-023-46484-3

**Published:** 2023-11-08

**Authors:** Fatemeh Tajik, Fahimeh Fattahi, Fereshteh Rezagholizadeh, Behnaz Bouzari, Pegah Babaheidarian, Masoud Baghai Wadji, Zahra Madjd

**Affiliations:** 1 Oncopathology Research Center, Iran University of Medical Sciences, Tehran, Iran; 2 Department of Surgery, University of California, Irvine, CA USA; 3 Clinical Research Development Unit of Ayatollah-Khansari Hospital, Arak University of Medical Sciences, Arak, Iran; 4 Department of Molecular Medicine, Faculty of Advanced Technologies in Medicine, Iran University of Medical Sciences, Tehran, Iran; 5 Department of Pathology, School of Medicine, Iran University of Medical Sciences, Tehran, Iran; 6 Department of Surgery, Firoozgar Hospital, Iran University of Medical Sciences, Tehran, Iran

**Keywords:** Cancer, Gastrointestinal cancer, Tumour biomarkers

## Abstract

DNA damage-inducible transcript 4 (DDIT4) is induced in various cellular stress conditions. Several studies showed that the dysregulation of DDIT4 is involved in different malignancies with paradoxical expressions and roles. Therefore, this study investigated the clinical significance, prognostic, and diagnostic value of DDIT4 in different types of pancreatic tumors (PT). The expression of DDIT4 and long non-coding RNA (TPTEP1) in mRNA level was examined in 27 fresh PT samples using Real-time quantitative PCR (RT-qPCR). Moreover, 200 formalin-fixed paraffin-embedded PT tissues, as well as 27 adjacent normal tissues, were collected to evaluate the clinical significance, prognostic, and diagnosis value of DDIT4 expression by immunohistochemistry (IHC) on tissue microarrays (TMA) slides. The results of RT-qPCR showed that the expression of DDIT4 in tumor samples was higher than in normal samples which was associated with high tumor grade (*P* = 0.015) and lymphovascular invasion (*P* = 0.048). Similar to this, IHC findings for nucleus, cytoplasm, and membrane localization showed higher expression of DDIT4 protein in PT samples rather than in nearby normal tissues. A statistically significant association was detected between a high level of nuclear expression of DDIT4 protein, and lymphovascular invasion (*P* = 0.025), as well as advanced TNM stage (*P* = 0.034) pancreatic ductal adenocarcinoma (PDAC) and in pancreatic neuroendocrine tumor (PNET), respectively. In contrast, a low level of membranous expression of DDIT4 protein showed a significant association with advanced histological grade (*P* = 0.011), margin involvement (*P* = 0.007), perineural invasion (*P* = 0.023), as well as lymphovascular invasion (*P* = 0.005) in PDAC. No significant association was found between survival outcomes and expression of DDIT4 in both types. It was found that DDIT4 has rational accuracy and high sensitivity as a diagnostic marker. Our results revealed a paradoxical role of DDIT4 expression protein based on the site of nuclear and membranous expression. The findings of this research indicated that there is a correlation between elevated nuclear expression of DDIT4 and the advancement and progression of disease in patients with PT. Conversely, high membranous expression of DDIT4 was associated with less aggressive tumor behavior in patients with PDAC. However, further studies into the prognostic value and biological function of DDIT4 are needed in future studies.

## Introduction

Pancreatic tumor (PT), a highly aggressive malignancy, is predicted to become the second leading cause of cancer-related deaths in the next decade^1^. Based on global cancer statistics 2020, the number of new cases and deaths of PT were 496,773 and 466,003, respectively, indicating an extremely fatal rate^2^. There were several histological types of PT comprising pancreatic ductal adenocarcinoma (PDAC), the most frequent, and pancreatic neuroendocrine tumor (PNET), which arise from pluripotent cells^3,4^. It has been found that the prevalence and incidence of PNET have gradually increased over the past 40 years and can be as high as 10% in autopsy studies^5,6^. The 5-year survival rate of PT is roughly 10 percent across the world, with minimal change throughout the past few decades^7,8^. Due to the early detection restrictions, low respectability rate at the time of diagnosis, early metastasis nature, and high resistance rate to neoadjuvant therapy, the prognosis is dismal^9,10^. Consequently, the need for prompt diagnosis and treatment of this insidious cancer is recognized globally. The discovery of tumor markers is a valuable and promising tool that has the potential in order to diagnose patients at an early stage of the disease, improve prognosis, and enhance treatment response^11–13^.

DNA damage-inducible transcript 4 (DDIT4), also known as a regulated in development and DNA damage response 1 (REDD1) protein or hypoxia-inducible factor 1 (HIF1)-responsive protein RTP801 (RTP801), was cloned in 2002^14,15^. It is strongly upregulated under various cellular stresses, such as hypoxia^16^, heat shock^17^, ionizing radiation^18^, and chemical molecules^19^. It has been indicated that DDIT4 inhibits the mammalian target of rapamycin complex 1 (mTORC1) and has a potential role in regulating cell growth, tumorigenesis, and autophagy pathway^20^.

A growing body of evidence has presented that dysregulation of DDIT4 occurs in diverse human cancers with contradictory roles as an oncogene or tumor suppressor^21–23^. As an oncogene, DDIT4 can cause the occurrence, and development of cancer via a link with RAS, stabilizing HIF1, and reducing apoptotic rate through increased anti-apoptotic proteins, which leads to an increase in cancer cell survival, proliferation, and migration and decreased apoptosis^22,24–30^. In accordance with this concept, an increase in the expression of DDIT4 at both the mRNA and protein levels has been observed in several types of cancer, such as colorectal carcinoma (CRC)^31,32^, bladder urothelial carcinoma (BUC)^22^, oral squamous cell carcinoma (OSCC)^33^, ovarian cancer (OC)^28,34^, and gastric cancer (GC)^27^. In contrast, several studies have demonstrated that DDIT4 is activated under diverse stress conditions in cells, resulting in cell death through inhibiting mTORC1^16,35–38^. Consequently, it appears that DDIT4 may act as a tumor-suppressing role in the progression of cancer^23,39–41^. Thus, the role of DDIT4 in cancer promotion or suppression is still unclear^42^.

Besides, in silico analysis indicated DDIT4 from the 3'-untranslated regions (UTR) binds to TPTE pseudogene 1 (TPTEP1), a long non-coding RNA (lncRNA) gene, regulates gene expression^31^. LncRNAs are non-coding RNAs with more than 200 nucleotides in length and have critical roles in existing cancers via gene transcription regulating, post-transcriptional protein activity control, or organizing nuclear domains^43–45^.

Hence, in the present study, the mRNA expression of DDIT4 and TPTEP1 in fresh PT samples and their adjacent normal tissues was assessed using RT-qPCR. Besides, for the first time, the expression levels and localization of DDIT4 protein were examined in the nucleus, cytoplasm, and membranous of PT tissues using the immunohistochemistry (IHC) method on tissue microarrays (TMAs) slides. Subsequently, the associations between expression levels of DDIT4 at different subcellular locations and clinicopathological features, also survival outcomes were analyzed.

## Results

### Bioinformatics approaches

**Figure 1:**
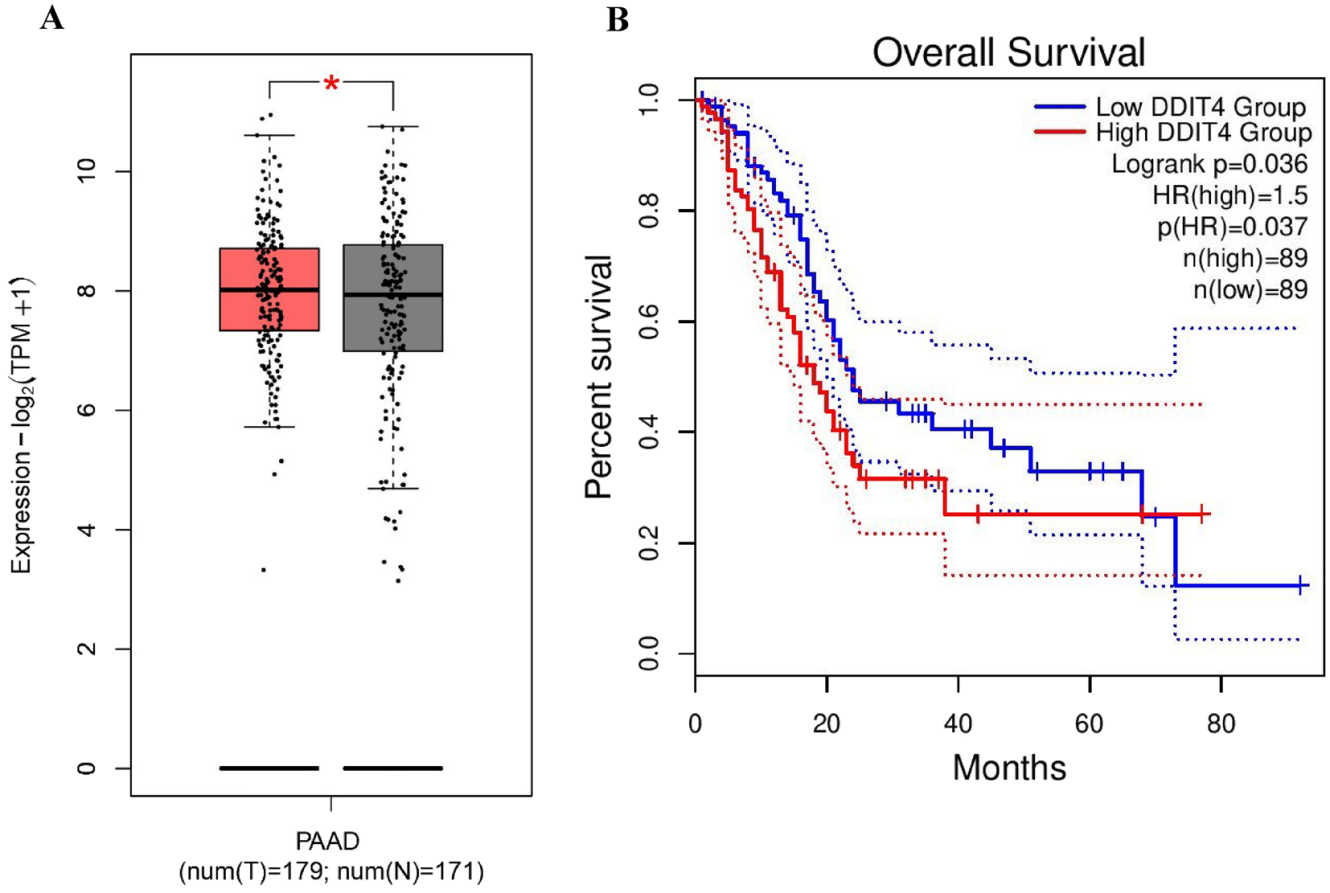
Box plot and overall survival (OS) results of DDIT4 expression for cancer compared to normal tissues through the Gene Expression Profiling Interactive Analysis (GEPIA2). The data through GEPIA2 indicated a few changes in the mRNA expression level of DDIT4 in pancreatic adenocarcinoma tissue samples compared to the normal tissues (*P* < 0.05, Fig. 1A). A high expression level of DDIT4 between the worse OS (HR (high) = 1.5, P (HR) = 0.037) and in PAAD patients (Fig. 1B).

Based on the analysis conducted on the TCGA database through GEPIA2, few changes were observed in the mRNA levels of DDIT4 in pancreatic adenocarcinoma (PAAD) tissue samples compared to the normal tissues (*P* < 0.05, Fig. 1A). Furthermore, the prognostic value of DDIT4 in PAAD was assessed by the use of transcriptome sequencing data from the GEPIA2 database. As a result, we found a high expression level of DDIT4 associated with the worse overall survival (OS) (HR (high) = 1.5, *P* (HR) = 0.037) in PAAD patients (Fig. 1B).

### Patients’ characteristics of fresh tissue samples

A total of 27 fresh tissue PDAC samples and 27 adjacent normal specimens were collected to investigate DDIT4 mRNA expression, of which 16 (59.2%) cases were males and 11 (40.8%) were females (male/female ratio = 1.45). The median age of patients was 62 ± 10.18 years old, ranging from 29 to 79, and the median tumor size was 2.7 cm (range: 1–12 cm). Histological grade of samples was available for 22 sample tissues, which are categorized into three groups as follows: 9 (40.9%) well-differentiated, 12 (54.5%) moderately differentiated, and 1 (4.6%) poorly differentiated. Further, samples were divided into three stages (I-III): 13 (56.5%) stage I, 8 (34.7%) stage II, and 2 (8.8%) stage III. Margin involvement, lymphovascular, and perineural invasion were observed in 3 (11.1%), 9 (33.3%), and 9 (33.3%), respectively. Lymph node metastasis was also present in 12 (44.4%) patients.

### Evaluation of DDIT4 and TPTEP1 mRNA expression level in fresh tumor tissues of pancreatic tumor and their adjacent normal tissues

**Figure 2:**
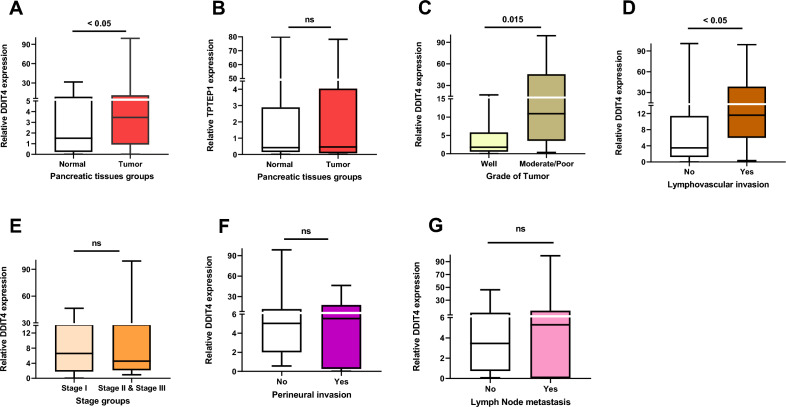
Boxplot of RT-qPCR data presented as median (min–max) for comparing expression levels of the **(A)** DDIT4 and **(B)** TPTEP1 genes between pancreatic tumor (PT) tissues and adjacent normal tissues; **(C)** Relationship between the expression of DDIT4 and histological grade; statistical significantly expression level of DDIT4 and increased with moderate/poor tumor histological grade. **(D)** Statistically increased expression level of DDIT4 was associated with lymphovascular invasion. Relationship between expression of DDIT4 gene and **(E)** stage, as well as **(F)** perineural invasion and **(G)** lymph node metastasis was not statistically significant.

The analysis of RT-qPCR data using the Mann–Whitney U test indicated that the mRNA expression levels of DDIT4 were significantly increased in PT patients compared to the healthy group (*P* < 0.05) (Fig. 2A). However, there was no significant difference between TPTEP1 mRNA expression in tumors and normal samples (Fig. 2B).

### Associations between DDIT4 mRNA expression and clinicopathological parameters in fresh tissue samples

Kruskal–Walli’s and Mann–Whitney U test analyses were performed to find any association between the median expression of the DDIT4 gene and the clinicopathological parameters of the PT patients. The findings of our study indicated a statistically significant increase in the median expression levels of DDIT4 in patients with moderate/poor tumor differentiation in comparison to those with well-differentiated tumors (*P* = 0.015) (Fig. 2C). Furthermore, as shown in frame D of Fig. 2, the median expression of DDIT4 was significantly higher in the patients with lymphovascular invasion than in patients without lymphovascular invasion (*P* = 0.048). There was no significant relationship between the median expression of DDIT4 and stage (I vs. II/III), as well as perineural invasion and lymph node metastasis (Fig. 2E–G).

### Characteristics of patients’ formalin-fixed paraffin-embedded (FFPE) tissue samples

To examine the clinical significance and subcellular localization of DDIT4 at the protein level, 200 FFPE tissue specimens were enrolled in this cross-sectional study, of whom 177 samples were PDAC, and the others were PNET type. The median age of total samples was 60 years old, ranging from 12 to 85. Among all patients, 108 (54.0%) cases were male, and 92 (46.0%) were female. The histological grade of tumor cells was divided into three categories as well, moderately, and weakly differentiated. All clinicopathological features of our total samples and types of PT are described in Table 1.

**Table 1:** Patients and tumor clinicopathological characteristic of pancreatic tumor and histological types of PTs.

Patients and tumor characteristics	Total samples N (%)	Pancreatic ductal adenocarcinoma (PDAC) N (%)	Pancreatic neuroendocrine tumor (PNET) N (%)
Number of samples	200	177	23
Median age, years (Range)	60 (12–85)	60 (50–85)	49 (12–74)
≤ Median age	103 (51.5)	87 (49.2)	12 (52.2)
> Median age	97 (48.5)	90 (50.8)	11 (47.8)
Sex			
Male	108 (54.0)	94 (53.1)	14 (60.9)
Female	92 (46.0)	83 (46.9)	9 (39.1)
Median tumor size (cm) (Range)	3 (0.3–16.5)	3 (0.3–16.5)	3 (0.4–10)
≤ Median	107 (53.5)	95 (53.7)	12 (52.2)
> Median	90 (45.0)	79 (44.6)	11 (47.8)
Histological grade			
Well-differentiated	81 (40.5)	65 (36.7)	16 (69.6)
Moderate differentiated	76 (38.0)	72 (40.7)	4 (17.4)
Poor differentiated	17 (8.5)	15 (8.5)	2 (8.7)
TNM stage			
I	59 (29.5)	55 (31.1)	4 (17.4)
II	80 (40.0)	77 (43.5)	3 (13.0)
III	19 (9.5)	19 (10.7)	0 (0.0)
IV	7 (3.5)	2 (1.1)	5 (21.7)
Tumor site			
Head	131 (65.5)	119 (67.2)	12 (52.2)
Body	10 (5.0)	9 (5.1)	1 (4.3)
Tail	1 (0.5)	1 (0.6)	0 (0.0)
Uncinate	54 (27.0)	44 (24.9)	10 (43.5)
Margin involvement			
Yes	36 (18.0)	34 (19.2)	2 (8.7)
No	137 (68.5)	127 (71.8)	10 (43.5)
Perineural invasion			
Present	103 (51.5)	100 (56.5)	3 (13.0)
Absent	65 (32.5)	58 (32.8)	7 (30.4)
Lymphovascular invasion			
Present	71 (35.5)	70 (39.5)	1 (4.3)
Absent	80 (40.0)	70 (39.5)	10 (43.5)
Lymph node (LN) metastasis			
Present	111 (55.5)	101 (57.1)	10 (43.5)
Absent	66 (33.0)	62 (35.0)	4 (17.4)
Macroscopic tumor extension			
Yes	124 (62.0)	113 (63.8)	11 (47.8)
No	47 (23.5)	41 (23.2)	6 (26.1)
Tumor recurrence			
Yes	35 (17.5)	33 (18.6)	2 (8.7)
No	155 (77.5)	137 (77.4)	18 (78.3)
Distant metastasis			
Yes	72 (36.0)	68 (38.4)	4 (17.4)
No	118 (59.0)	102 (57.6)	16 (69.6)

### Expression of DDIT4 in pancreatic tumor compared with adjacent normal samples

**Figure 3:**
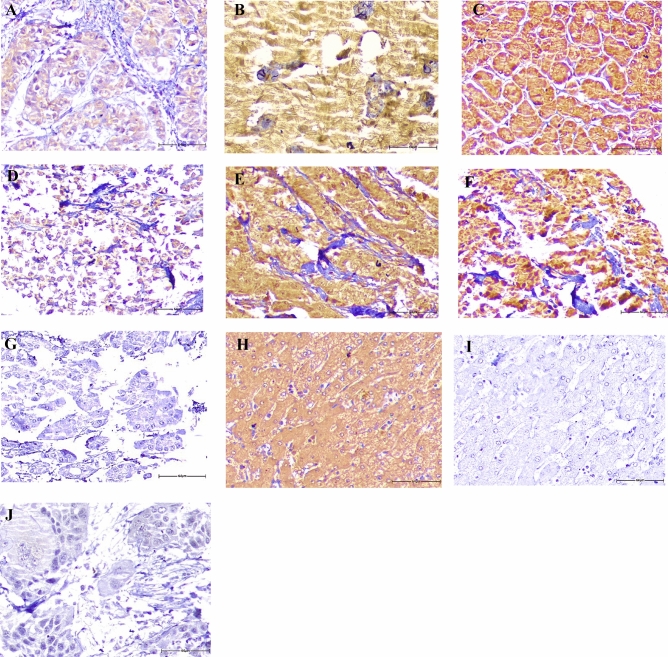
Immunohistochemical analysis of DDIT4 protein expression in pancreatic samples. DDIT4 expression in pancreatic ductal adenocarcinoma: **A** low expression, **B** moderate expression, **C** high expression. In pancreatic neuroendocrine tumor: **D** low expression, **E** moderate expression **F** high expression. IHC staining of (**G**) adjacent normal tissue, normal liver tissues as (**H**) positive and (**I**) negative controls, and also (**J**) isotype controls (Figures shown with a magnification of 400 ×).

**Figure 4:**
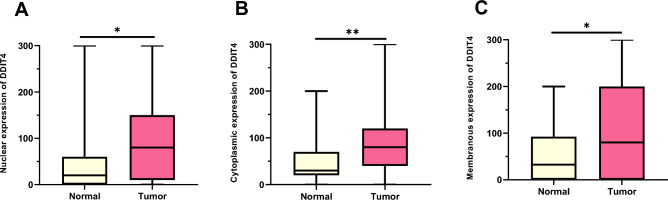
Box plot analysis of DDIT4 expression levels in tumor and normal tissue samples. The result of data analysis showed a statistically significant association between the **(A)** nuclear, **(B)** cytoplasmic, and **(C)** membranous expression of tumor and normal samples.

The protein expression levels of DDIT4 were evaluated using the IHC technique on TMA sections by three distinct scoring methods that comprise the intensity of staining, percentage of positive tumor cells, and H-score. DDIT4 was expressed at different intensities in the nucleus, cytoplasm, and membranous in two types of PT as well as adjacent normal samples (Fig. 3). The mean expression level (based on H-score) of DDIT4 was 80 in tumor samples (in all expression); however, the mean expression of DDIT4 was 20 in the nucleus, 30 in the cytoplasm, and 32 in the membranous in adjacent normal tissue, indicating that the expression of DDIT4 in PTs was high compared to adjacent normal tissues (Table 2 and Fig. 4). Besides, Mann–Whitney U tests showed a significant difference between the median nuclear, cytoplasmic, and membranous expression of DDIT4, and the median expression of adjacent normal tissues (*P* = 0.012, *P* = 0.001, and *P* = 0.026, respectively) (Table 2).

**Table 2:** Association of nuclear, cytoplasmic, membranous DDIT4 protein expressions (Intensity of staining, percentage of positive tumor cells, and H-score) between types of pancreatic tumor and benign tumors.

Expression of DDIT4	Nuclear expression				
	Total samples N (%)	Pancreatic ductal adenocarcinoma (PDAC) N (%)	Pancreatic neuroendocrine tumor (PNET) N (%)	Adjacent normal tissues N (%)	*P value*
Intensity of staining					
Negative (0)	45 (22.5)	40 (22.6)	5 (21.7)	11 (39.3)	***0.038***
Weak (+ 1)	58 (29.0)	53 (29.9)	5 (21.7)	8 (28.6)	
Moderate (+ 2)	63 (31.5)	54 (30.5)	9 (39.1)	7 (25.0)	
Strong (+ 3)	34 (17.0)	30 (16.9)	4 (17.4)	2 (7.1)	
Percentage of positive tumor cells					
< 25%	66 (33.0)	59 (33.3)	7 (30.4)	16 (57.1)	***0.024***
25–50%	41 (20.5)	37 (20.9)	4 (17.4)	5 (17.9)	
51–75%	30 (15.0)	28 (15.8)	2 (8.7)	1 (3.6)	
> 75%	63 (31.5)	53 (20.9)	10 (43.5)	6 (21.4)	
H-score cut-off	80	62	100	20	
Low	109 (54.5)	88 (49.7)	13 (56.5)	15 (53.6)	***0.012***
High	91 (45.5)	89 (50.3)	10 (43.5)	13 (46.4)	
Expression of DDIT4	Cytoplasmic expression				
Intensity of staining					
Negative (0)	18 (9.0)	14 (7.9)	4 (17.4)	5 (17.9)	***0.002***
Weak (+ 1)	96 (48.0)	84 (47.5)	12 (52.2)	19 (67.9)	
Moderate (+ 2)	67 (33.5)	60 (33.9)	7 (30.4)	4 (14.3)	
Strong (+ 3)	19 (9.5)	19 (10.7)	0 (0.0)	0 (0.0)	
Percentage of positive tumor cells					
< 25%	33 (16.5)	28 (15.8)	5 (21.7)	10 (35.7)	***0.02***
25–50%	70 (35.0)	59 (33.3)	11 (47.8)	11 (39.3)	
51–75%	29 (14.5)	27 (15.3)	2 (8.7)	0 (0.0)	
> 75%	68 (34.0)	63 (35.6)	5 (21.7)	7 (25.0)	
H-score cut-off	80	80	50	30	
Low	115 (57.5)	98 (55.4)	13 (56.5)	18 (64.3)	***0.001***
High	85 (42.5)	79 (44.6)	10 (43.5)	10 (35.7)	
Expression of DDIT4	Membranous expression				
Intensity of staining					
Negative (0)	64 (32.0)	56 (31.6)	8 (34.8)	11 (39.3)	***0.035***
Weak (+ 1)	56 (28.0)	50 (28.2)	6 (26.1)	13 (46.4)	
Moderate (+ 2)	39 (19.5)	32 (18.1)	7 (30.4)	3 (10.7)	
Strong (+ 3)	41 (20.5)	39 (22.0)	2 (8.7)	1 (3.6)	
Percentage of positive tumor cells					
< 25%	75 (37.5)	66 (37.3)	9 (39.1)	15 (53.6)	***0.039***
25–50%	21 (10.5)	17 (9.6)	4 (17.4)	2 (7.1)	
51–75%	13 (6.5)	11 (6.2)	2 (8.7)	5 (17.9)	
> 75%	91 (45.5)	83 (46.9)	8 (34.8)	6 (21.4)	
H-score cut-off	80	85	50	32	
Low	102 (51.0)	88 (49.7)	12 (52.2)	14 (50.0)	***0.026***
High	98 (49.0)	89 (50.3)	11 (47.8)	14 (50.0)	

*P value*; Kruskal–Wallis & Mann–Whitney U tests.

H-score; histological score.

Values in bold and italic are statistically significant.

### Associations between DDIT4 expression (nucleus, cytoplasm, and membrane) and clinicopathological characteristics of PDAC tissue samples

The association between the expression of DDIT4 protein and the clinicopathological parameters of PT was investigated using Pearson's χ2 test. Our analysis demonstrated a significant association between the nuclear expression of DDIT4 protein and lymphovascular invasion (*P* = 0.025). We also found a statistically significant association between a membranous expression of DDIT4 protein and histological grade (*P* = 0.011), margin involvement (*P* = 0.007), perineural invasion (*P* = 0.023), as well as a lymphovascular invasion (*P* = 0.005). There was no significant association between cytoplasmic expression of DDIT4 and clinicopathological features (Table 3). Besides, the correlation between DDIT4 protein expression and clinicopathological characteristics was examined using spearman’s correlation tests. Our results indicated a significant direct correlation between a high level of nuclear expression of DDIT4 and lymphovascular invasion (*P* = 0.009). Furthermore, we observed an inverse correlation between increased membranous expression of DDIT4 protein and advanced histological grade (*P* = 0.011), margin involvement (*P* = 0.001), perineural invasion (*P* = 0.007), as well as lymphovascular invasion (*P* = 0.001).

**Table 3:** The association between expression of DDIT4 and clinicopathological features of pancreatic ductal adenocarcinoma carcinoma (PDAC) (*P value*; Pearson’s χ2 test).

Tumor characteristics	Total samples N (%)	Nuclear expression		*P value*	Cytoplasmic expression		*P value*	Membranous expression		*P value*
		H score (cut off = 62) N %			H score (cut off = 80) N %			H score (cut off = 85) N %		
		Low (≤ 62)	High (> 62)		Low (≤ 80)	High (> 80)		Low (≤ 85)	High (> 85)	
Number of samples	177	88	89		98	79		88	89	
Median age, years (Range)	60 (50–85)									
≤ Median age	87 (49.2)	39 (44.3)	48 (53.9)	0.201	46 (46.9)	41 (51.9)	0.512	38 (43.2)	49 (55.1)	0.114
> Median age	90 (50.8)	49 (55.7)	41 (46.1)		52 (53.1)	38 (48.1)		50 (56.8)	40 (44.9)	
Sex										
Male	94 (53.1)	48 (54.5)	46 (51.7)	0.703	51 (52.0)	43 (54.4)	0.751	53 (60.2)	41 (46.1)	0.059
Female	83 (46.9)	40 (45.5)	43 (48.3)		47 (48.0)	36 (45.6)		35 (39.8)	48 (53.9)	
Median tumor size (cm) (Range)	3 (0.3–16.5)									
≤ Median	95 (53.7)	44 (50.6)	51 (58.6)	0.286	49 (51.0)	46 (59.0)	0.296	48 (55.2)	47 (54.0)	0.879
> Median	79 (44.6)	43 (49.4)	36 (41.4)		47 (49.0)	32 (41.0)		39 (44.8)	40 (46.0)	
Histological grade										
Well-differentiated	65 (36.7)	29 (33.0)	36 (40.4)	0.138	39 (39.8)	26 (32.9)	0.322	30 (34.1)	35 (39.3)	***0.011***
Moderate differentiated	72 (40.7)	33 (37.5)	39 (43.8)		34 (34.7)	38 (48.1)		29 (33.0)	43 (48.3)	
Poor differentiated	15 (8.5)	11 (12.5)	4 (4.5)		10 (10.2)	5 (6.3)		11 (12.5)	4 (4.5)	
TNM stage										
I	55 (31.1)	30 (34.1)	25 (28.1)		34 (34.7)	21 (26.6)	0.083	27 (30.7)	28 (31.5)	
II	77 (43.5)	36 (40.9)	41 (46.1)	0.599	43 (43.9)	34 (43.0)		37 (42.0)	40 (44.9)	0.261
III	19 (10.7)	7 (8.0)	12 (13.5)		6 (6.1)	13 (16.5)		8 (9.1)	11 (12.4)	
IV	2 (1.1)	1 (1.1)	1 (1.1)		0 (0.0)	2 (2.5)		0 (0.0)	2 (2.2)	
Tumor site										
Head	119 (67.2)	61 (69.3)	58 (65.2)	0.269	65 (66.3)	54 (68.4)		59 (67.0)	60 (67.4)	0.204
Body	9 (5.1)	5 (5.7)	4 (4.5)		5 (5.1)	4 (5.1)	0.632	4 (4.5)	5 (5.6)	
Tail	1 (0.6)	0 (0.0)	1 (1.1)		1 (1.0)	0 (0.0)		0 (0.0)	1 (1.1)	
Uncinate	44 (24.9)	22 (25.0)	22 (24.7)		26 (26.5)	18 (22.8)		25 (28.4)	19 (21.3)	
Margin involvement										
Yes	34 (19.2)	17 (19.3)	17 (19.1)	0.854	14 (14.3)	20 (25.3)	0.125	10 (11.4)	24 (27.0)	***0.007***
No	127 (71.8)	62 (70.5)	65 (73.0)		73 (74.5)	54 (68.4)		66 (75.0)	61 (68.5)	
Perineural invasion										
Present	100 (56.5)	46 (52.3)	54 (60.7)	0.364	50 (51.0)	50 (63.3)	0.064	41 (46.6)	59 (66.3)	***0.023***
Absent	58 (32.8)	30 (34.1)	28 (31.5)		33 (33.7)	25 (31.6)		34 (38.6)	24 (27.0)	
Lymphovascular invasion										
Present	70 (39.5)	28 (31.8)	42 (47.2)	***0.025***	35 (35.7)	35 (44.3)	0.507	26 (29.5)	44 (49.4)	***0.005***
Absent	70 (39.5)	35 (39.8)	35 (39.3)		41 (41.8)	29 (36.7)		36 (40.9)	34 (38.2)	
Lymph node (LN) metastasis										
Present	101 (57.1)	45 (51.1)	56 (62.9)	0.233	52 (53.1)	49 (62.0)	0.465	45 (51.1)	56 (62.9)	0.054
Absent	62 (35.0)	34 (38.6)	28 (31.5)		37 (37.8)	25 (31.6)		32 (36.4)	30 (33.7)	
Macroscopic tumor extension										
Yes	113 (63.8)	48 (54.5)	65 (73.0)	0.166	59 (60.2)	54 (68.4)	0.31	48 (54.5)	65 (73.0)	0.124
No	41 (23.2)	24 (27.3)	17 (19.1)		23 (23.5)	18 (22.8)		24 (27.3)	17 (19.1)	
Tumor recurrence										
Yes	33 (18.6)	16 (19.5)	17 (19.3)	0.975	17 (18.1)	16 (21.1)	0.627	17 (20.2)	16 (18.6)	0.788
No	137 (77.4)	66 (80.5)	71 (80.7)		77 (81.9)	60 (78.9)		67 (79.8)	70 (81.4)	
Distant metastasis										
Yes	68 (38.4)	34 (41.5)	34 (38.6)	0.707	42 (44.7)	26 (34.2)	0.166	35 (41.7)	33 (38.4)	0.661
No	102 (57.6)	48 (58.5)	54 (61.4)		52 (55.3)	50 (65.8)		49 (58.3)	53 (61.6)	

P value; Pearson's chi-square.

H-score; histological score.

Values in bold and italic are statistically significant.

### Associations between DDIT4 expression (nucleus, cytoplasm, and membrane) and clinicopathological characteristics of PNET tissue samples

Pearson's χ2 test exhibited a significant association between the nuclear expression of DDIT4 protein and histological grade (*P* = 0.034). Moreover, our Spearman’s correlation tests showed a direct correlation between the nuclear expression of DDIT4, and the high grade (*P* = 0.014). We did not find any association or correlation between cytoplasmic and membranous DDIT4 protein expressions and the clinicopathological parameters (Table 4).

**Table 4:** The association between expression of DDIT4 and clinicopathological features of pancreatic neuroendocrine tumor (PNET) (*P value*; Pearson’s χ2 test).

Tumor characteristics	Total samples N (%)	Nuclear expression		*P value*	Cytoplasmic expression		*P value*	Membranous expression		*P value*
		H score (cut off = 100) N %			H score (cut off = 50) N %			H score (cut off = 50) N %		
		Low (≤ 100)	High (> 100)		Low (≤ 50)	High (> 50)		Low (≤ 50)	High (> 50)	
Number of samples	23	13	10		13	10		12	11	
Median age, years (Range)	49 (12–74)									
≤ Median age	12 (52.2)	7 (53.8)	5 (50.0)	855	5 (38.5)	7 (70.0)	0.133	6 (50.0)	6 (54.5)	0.827
> Median age	11 (47.8)	6 (46.2)	5 (50.0)		8 (61.5)	3 (30.0)		6 (50.0)	5 (45.5)	
Sex										
Male	14 (60.9)	7 (53.8)	7 (70.0)	0.431	11 (84.6)	3 (30.0)	0.067	6 (50.0)	8 (72.7)	0.265
Female	9 (39.1)	6 (46.2)	3 (30.0)		2 (15.4)	7 (70.0)		6 (50.0)	3 (27.3)	
Median tumor size (cm) (Range)	3 (0.4–10)									
≤ Median	12 (52.2)	8 (61.5)	4 (40.0)	0.305	7 (53.8)	5 (50.0)	0.855	7 (58.3)	5 (45.5)	0.537
> Median	11 (47.8)	5 (38.5)	6 (60.0)		6 (46.2)	5 (50.0)		5 (41.7)	6 (54.5)	
Histological grade										
Well-differentiated	16 (69.6)	10 (76.9)	6 (60.0)	***0.034***	7 (53.8)	9 (90.0)	0.176	8 (66.7)	8 (72.7)	0.811
Moderate differentiated	4 (17.4)	2 (15.4)	2 (20.0)		4 (30.8)	0 (0.0)		2 (16.7)	2 (18.2)	
Poor differentiated	2 (8.7)	1 (7.7)	1 (10.0)		1 (7.7)	1 (10.0)		1 (8.3)	1 (9.1)	
TNM stage										
I	4 (17.4)	4 (30.8)	0 (0.0)	0.651	1 (7.7)	3 (30.0)	0.324	4 (33.3)	0 (0.0)	0.103
II	3 (13.0)	3 (23.1)	0 (0.0)		2 (15.4)	1 (10.0)		2 (16.7)	1 (9.1)	
III	0 (0.0)	0 (0.0)	0 (0.0)		2 (15.4)	3 (30.0)		0 (0.0)	0 (0.0)	
IV	5 (21.7)	1 (7.7)	4 (40.0)		8 (61.5)	3 (30.0)		1 (8.3)	4 (36.4)	
Tumor site										
Head	12 (52.2)	8 (61.5)	4 (40.0)	0.372	8 (61.5)	4 (40.0)	0.372	8 (66.7)	4 (36.4)	0.26
Body	1 (4.3)	0 (0.0)	1 (10.0)		0 (0.0)	1 (10.0)		0 (0.0)	1 (9.1)	
Tail	0 (0.0)	0 (0.0)	0 (0.0)		0 (0.0)	0 (0.0)		0 (0.0)	0 (0.0)	
Uncinate	10 (43.5)	5 (38.5)	5 (50.0)		5 (38.5)	5 (50.0)		4 (33.3)	6 (54.5)	
Margin involvement										
Yes	2 (8.7)	1 (7.7)	1 (10.0)	0.951	2 (15.4)	0 (0.0)	0.237	1 (8.3)	1 (9.1)	0.799
No	10 (43.5)	6 (46.2)	4 (40.0)		4 (30.8)	6 (60.0)		6 (50.0)	4 (36.4)	
Perineural invasion										
Present	3 (13.0)	2 (15.4)	1 (10.0)	0.673	1 (7.7)	2 (20.0)	0.673	2 (16.7)	1 (9.1)	0.321
Absent	7 (30.4)	3 (23.1)	4 (40.0)		4 (30.8)	3 (30.0)		5 (41.7)	2 (18.2)	
Lymphovascular invasion										
Present	1 (4.3)	0 (0.0)	1 (10.0)	0.505	1 (7.7)	0 (0.0)	0.304	0 (0.0)	1 (9.1)	0.052
Absent	10 (43.5)	6 (46.2)	4 (40.0)		4 (30.8)	6 (60.0)		8 (66.7)	2 (18.2)	
Lymph node (LN) metastasis										
Present	10 (43.5)	7 (53.8)	3 (30.0)	0.307	4 (30.8)	6 (60.0)	0.242	5 (41.7)	5 (45.5)	0.586
Absent	4 (17.4)	1 (7.7)	3 (30.0)		2 (15.4)	2 (20.0)		3 (25.0)	1 (9.1)	
Macroscopic tumor extension										
Yes	11 (47.8)	5 (38.5)	6 (60.0)	0.591	6 (46.2)	5 (50.0)	0.83	7 (58.3)	4 (36.4)	0.486
No	6 (26.1)	4 (30.8)	2 (20.0)		3 (23.1)	3 (30.0)		2 (16.7)	4 (36.4)	
Tumor recurrence										
Yes	2 (8.7)	0 (0.0)	2 (22.2)	0.099	1 (9.1)	1 (11.1)	0.881	2 (18.2)	0 (0.0)	0.178
No	18 (78.3)	11 (100.0)	7 (77.8)		10 (90.9)	8 (88.9)		9 (81.8)	9 (100.0)	
Distant metastasis										
Yes	4 (17.4)	1 (9.1)	3 (33.3)	0.178	1 (9.1)	3 (33.3)	0.178	2 (18.2)	2 (22.2)	0.822
No	16 (69.6)	10 (90.0)	6 (66.7)		10 (90.9)	6 (66.7)		9 (81.8)	7 (77.8)	

P value; Pearson's chi-square.

H-score; histological score.

Values in bold and italic are statistically significant.

### Information on clinical outcomes in types of PT

In the current study, from 200 patients, follow-up data for 10 patients were not available. Of them, 7 patients had PDAC, and 3 patients had PNET.

#### Pancreatic ductal adenocarcinoma cancer

Among 190 patients, 33 (19.4%) showed tumor recurrence, 68 (40.0%) had metastasis, and 8 (4.7%) showed recurrence and metastasis. Cancer-related death occurred in 106 (62.3%) patients. The mean OS/disease-specific survival (DSS) and progression-free survival (PFS) follow-up time for patients with high nuclear expression was shorter than the low nuclear expression of DDIT4 (OS/DSS = 17 and 20 months, respectively, and PFS = 13 and 16 months, respectively). However, the mean OS/DSS and PFS follow-up time for patients with high cytoplasmic, and membranous expression were longer than the low expression of DDIT4 (cytoplasmic: OS/DSS = 20 and 17 months, and PFS = 16 and 13 months, respectively; membranous: OS/DSS = 19 and 18 months, and PFS = 15 and 14 months, respectively); indicating that high nuclear expression has shorter survival than low expression; while high cytoplasmic and membranous expression of DDIT4 was related to longer survival. The main characteristics of survival outcomes are exhibited in Table 5.

**Table 5:** The main characteristics of patients’ survival analysis based on types of pancreatic tumor (PT).

Features	Total samples	Histological types of PT	
		PDAC	PNET
Number of patients (N)	190	170	20
Range of follow-up (month)	0–82	0–82	1–50
Mean duration of follow-up time (month) for OS, DSS (SD)	19 (16.19), 19 (16.29)	18 (16.33), 19 (16.47)	27 (12.92), 27 (12.92)
Median duration of follow-up time (month) for OS, DSS (Q1, Q3)	17 (6, 30), 17 (6, 30)	15 (5, 27), 15 (6, 29)	27 (18, 37), 27 (18, 37)
Mean duration of follow-up time (month) for PFS (SD)	16 (16.24)	15 (16.23)	24 (14.03)
Median duration of follow-up time (month) for PFS (Q1, Q3)	12 (2, 26)	9 (2, 24)	25 (17, 35)
Cancer-related death (N %)	113 (56.5)	106 (62.3)	7 (35.0)
Death due to other reasons (N %)	7 (3.5)	7 (4.1)	0 (0.0)
Distant metastasis during follow-up (N %)	72 (36.0)	68 (40.0)	4 (20.0)
Tumor recurrence during follow-up (N %)	35 (17.5)	33 (19.4)	2 (10.0)
Alive patients without distant metastasis and tumor recurrence (N %)	58 (63.0)	47 (27.6)	11 (55.5)

#### Pancreatic neuroendocrine tumor

In this type of PT, tumor recurrence and metastasis occurred in 2 (10.0) and 4 (20.0) patients, respectively. Throughout follow-up, all death cases died of pancreatic tumor. The median and mean OS and DSS time were 27 months (Q1 = 18 and Q3 = 37) and 27 (SD = 12.92), respectively. Additionally, the median and mean follow-up periods for PFS were 25 (Q1 = 17, Q3 = 35) and 24 (SD = 14.03) months, respectively. Patients with high nuclear DDIT4 expression have been reported to have lower mean OS, DSS and PFS survival, which is consistent with PDAC survival data. However, there is not any difference between the mean OS/DSS/PFS and high/low cytoplasmic and membranous expression.

### Survival analysis based on the expression of DDIT4 in PT types

#### Pancreatic ductal adenocarcinoma cancer

**Figure 5:**
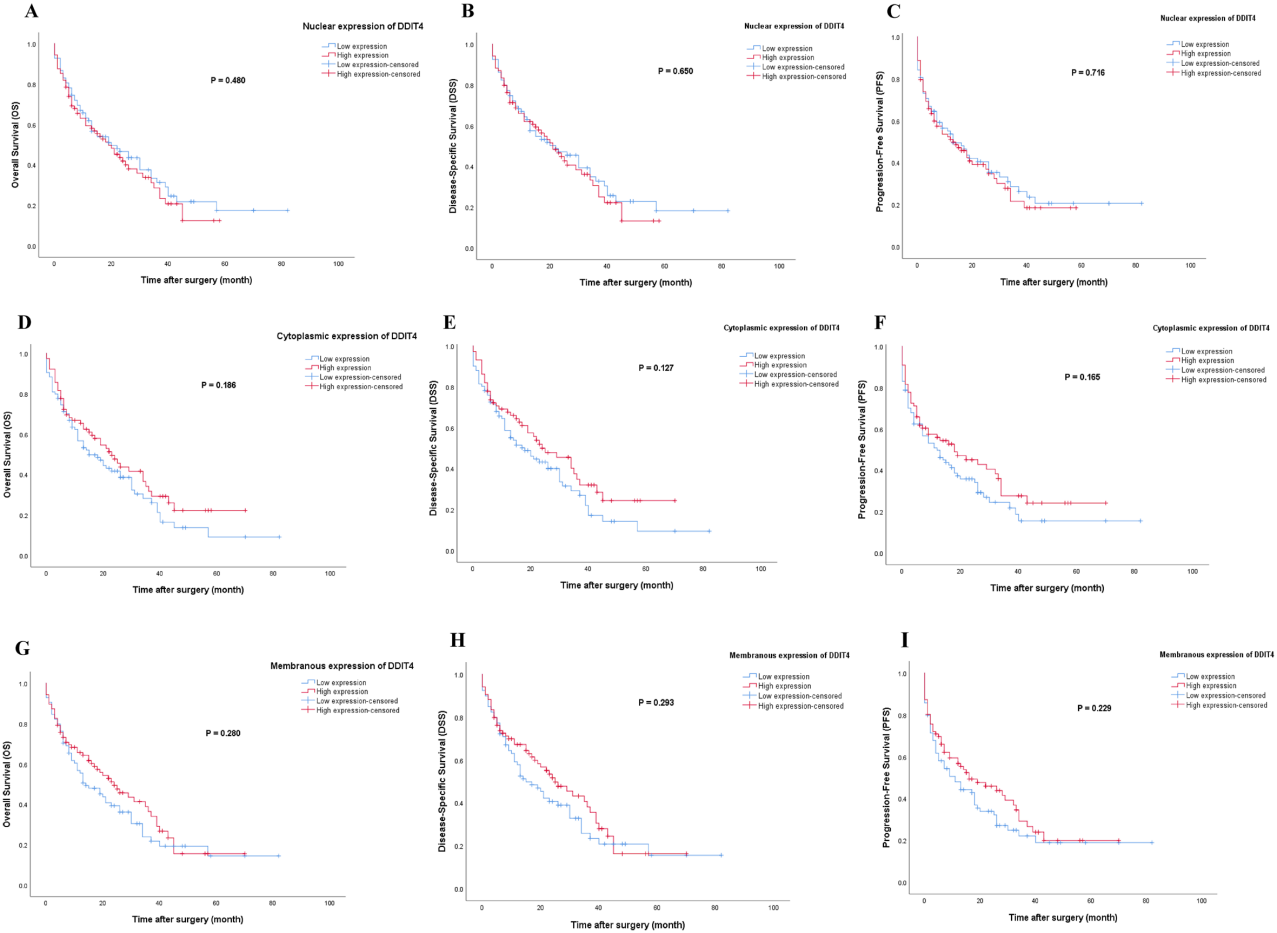
Kaplan–Meier survival curves for overall survival (OS), disease-specific survival (DSS), and progression-free survival (PFS) based on nuclear, cytoplasmic, and membranous DDIT4 protein expression levels in pancreatic ductal adenocarcinoma (PDAC). The Kaplan–Meier survival analysis showed no significant differences between OS, DSS or PFS and the patients with high and low nuclear (**A, B, C**), cytoplasmic (**D, E, F**), and membranous (**G, H, I**) expression of DDIT4 protein.

Kaplan–Meier survival analysis exhibited no statistically significant differences between OS/DSS/PFS and the patients with high /low expression of DDIT4 (Fig. 5).

Univariate and multivariate Cox regression analyses were performed to evaluate the clinical significance of potential prognostic factors for OS, DSS, and PFS. On univariate analyses, tumor size, TNM stage, and distant metastasis were detected as potential prognostic factors for OS, DSS, and PFS. In addition, age and lymphovascular invasion have a prognostic role for OS/PFS and DSS, respectively. Statistically significant univariate analyses were incorporated into multivariate Cox regression analysis, in which the results indicated that distant metastasis was an independent risk factor affecting OS (HR: 187; 95% CI 1.479–3.233; *P* < 0.001), DSS (HR: 2.286; 95% CI 1.528–3.421; *P* < 0.001), and PFS (HR: 2.680; 95% CI 1.819–3.949; *P < 0.001*, respectively) (Table 6).

**Table 6:** Univariate and multivariate cox regression analyses of potential prognostic factors for Overall Survival (OS), Disease-Specific survival (DSS), and Progression-Free Survival (PFS) in patients with pancreatic ductal adenocarcinoma (PDAC).

Covariate	Overall Survival (OS)				Disease-Specific survival (DSS)				Progression-Free Survival (PFS)			
	Univariate analysis		Multivariate analysis		Univariate analysis		Multivariate analysis		Univariate analysis		Multivariate analysis	
	HR (95% CI)	*P-value*	HR (95% CI)	*P-value*	HR (95% CI)	*P-value*	HR (95% CI)	*P-value*	HR (95% CI)	*P-value*	HR (95% CI)	*P-value*
**Age (years)**	1.503 (1.036–2.182)	***0.032***	1.479 (0.990–2.209)	0.056	1.450 (0.987–2.132)	0.058	–	–	1.463 (1.008–2.123)	***0.045***	1.405 (0.947–2.085)	0.091
**Tumor size (cm)**	1.543 (1.059–2.247)	***0.024***	1.430 (0.968–2.113)	0.073	1.540 (1.042–2.275)	***0.03***	1.468 (0.980–2.198)	0.062	1.519 (1.044–2.210)	***0.029***	1.470 (0.993–2.177)	0.055
**Histological grade**		0.731	–	–		0.638	–	–		0.879	–	–
Moderate versus well	0.757 (0.440–1.303)	0.315			0.712 (0.406–1.249)	0.236			0.819 (0.476–1.409)	0.47		
Poor versus well	0.762 (0.444–1.309)	0.325			0.756 (0.433–1.320)	0.325			0.877 (0.511–1.506)	0.635		
**TNM stage**		***0.008***		0.167		***0.005***		0.104		***0.005***		0.099
I versus II	0.389 (0.216–0.702)	***0.002***	0.547 (0.292–1.025)	0.06	0.354 (0.192–0.655)	***0.001***	0.517 (0.266–1.004)	0.051	0.439 (0.244–0.790)	***0.006***	0.596 (0.319–1.112)	0.104
I versus III	0.633 (0.372–1.080)	0.093	0.686 (0.389–1.209)	0.192	0.620 (0.358–1.073)	0.087	0.812 (0.436–1.514)	0.513	0.690 (0.405–1.173)	0.171	0.732 (0.415–1.290)	0.28
I versus IV	0.745 (0.377–1.470)	0.396	0.827 (0.395–1.729)	0.613	0.706 (0.350–1.424)	0.333	0.832 (0.375–1.845)	0.652	0.855 (0.434–1.685)	0.651	0.962 (0.466–1.986)	0.916
**Margin Involvement**	1.091 (0.750–1.588)	0.648	–	–	1.031 (0.703–1.513)	0.876	–	–	0.924 (0.633–1.350)	0.684	–	–
**Perineural invasion**	1.151 (0.872–1.518)	0.321	–	–	1.165 (0.876–1.550)	0.294	–	–	1.057 (0.803–1.391)	0.692	–	–
**Lymphovascular invasion**	1.272 (0.984–1.645)	0.066	–	–	1.320 (1.012–1.720)	***0.04***	1.307 (0.983–1.736)	0.065	1.250 (0.969–1.612)	0.086	–	–
**Lymph node (LN) metastasis**	1.105 (0.821–1.489)	0.51	–	–	1.106 (0.813–1.505)	0.521	–	–	1.124 (0.840–1.504)	0.432	–	–
**Macroscopic tumor extension**	0.997 (0.775–1.284)	0.984	–	–	1.012 (0.782–1.311)	0.927	–	–	0.965 (0.747–1.245)	0.783	–	–
**Tumor recurrence**	0.800 (0.516–1.242)	0.321	–	–	0.800 (0.507–1.264)	0.34	–	–	0.661 (0.425–1.029)	0.067	–	–
**Distant metastasis**	2.229 (1.530–3.248)	*** < 0.001***	2.187 (1.479–3.233)	*** < 0.001***	2.427 (1.639–3.594)	*** < 0.001***	2.286 (1.528–3.421)	*** < 0.001***	2.651 (1.816–3.880)	*** < 0.001***	2.680 (1.819–3.949)	*** < 0.001***
**Nuclear DDIT4 expression**												
High versus low	1.140 (0.787–1.652)	0.488	–	–	1.091 (0.743–1.603)	0.656	–	–	1.069 (0.738–1.547)	0.725	–	–
**Cytoplasmic DDIT4 expression**												
High versus low	0.780 (0.536–1.135)	0.194	–	–	0.742 (0.501–1.097)	0.135	–	–	0.773 (0.531–1.126)	0.18	–	–
**Membranous DDIT4 expression**												
High versus low	0.819 (0.565–1.185)	0.289	–	–	0.817 (0.557–1.199)	0.302	–	–	0.803 (0.554–1.162)	0.244	–	–

*HR;* hazard ratio, *CI*; confidence interval.

The variables with *P* value less than 0.05 were included in multivariable analyses.

Values in bold and italic are statistically significant.

#### Pancreatic neuroendocrine tumor

Kaplan–Meier survival analysis indicated that DDIT4 expression was not a prognostic factor in OS (Log-rank test; nuclear: *P* = 0.812, cytoplasmic: *P* = 0.955, membranous: *P* = 0.603), DSS (Log-rank test; nuclear: *P* = 0.812, cytoplasmic: *P* = 0.955, membranous: *P* = 0.603), and PFS (Log-rank test; nuclear: *P* = 0.563, cytoplasmic: *P* = 0.967, membranous: *P* = 0.524). Besides, the results of univariate and multivariate Cox regression analyses showed that the clinicopathologic parameters were not significant factors affecting OS, DSS, or PFS in the patients with PNET.

### Diagnostic value of the DDIT4 in PT versus adjacent normal tissues

**Figure 6:**
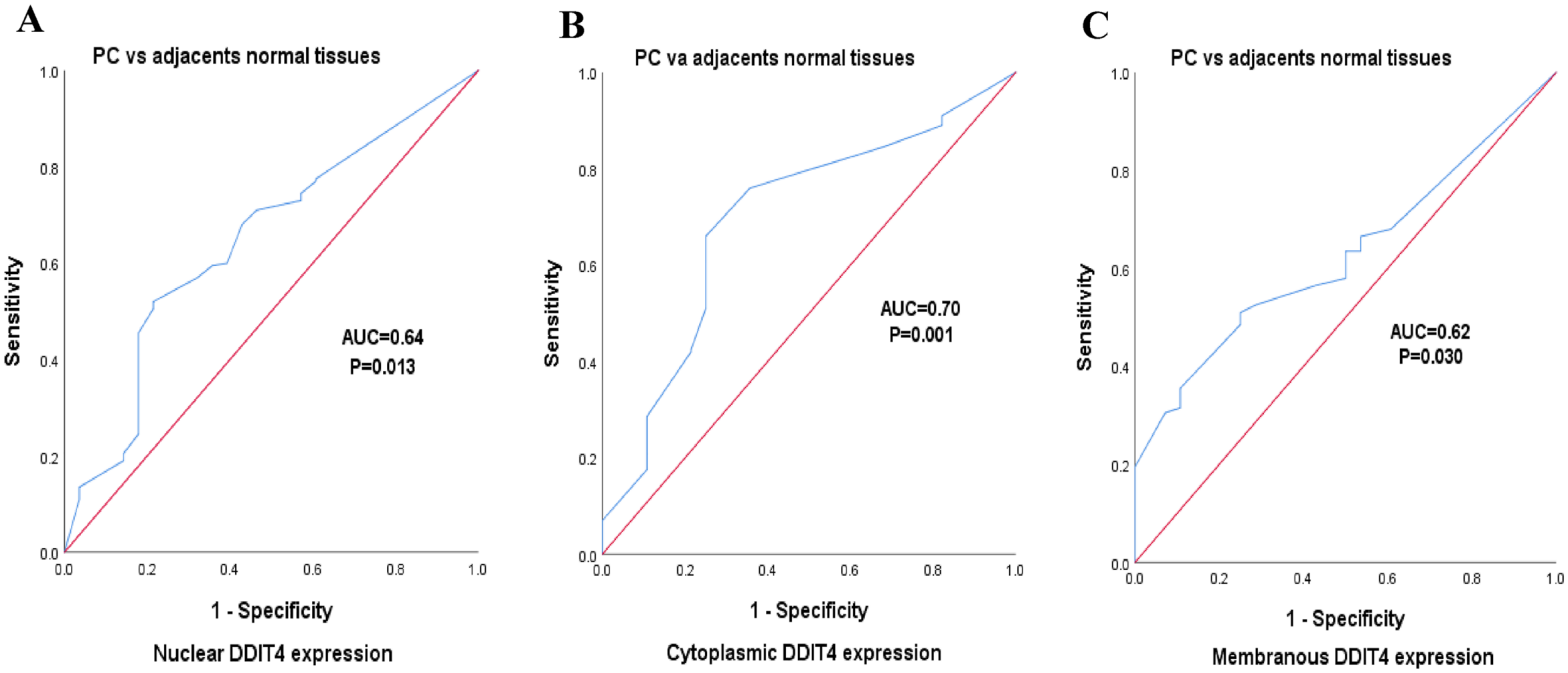
Receiver operating characteristic (ROC) curves analysis between nuclear, cytoplasmic, and membranous expression of DDIT4 in PT and adjacent normal tissues with immunohistochemical analysis. (**A**) The results showed that an AUC of 0.64, 95% CI 0.53–0.75, *P* = 0.013 for nuclear DDIT4 expression in PT vs adjacent normal tissues. (**B**) AUC of 0.70, 95% CI 0.59–0.80, *P* = 0.001 for cytoplasmic DDIT4 expression in PT vs adjacent normal tissues. (**C**) AUC of 0.62, 95% CI 0.53–0.71, *P* = 0.030 for membranous DDIT4 expression in PT vs adjacent normal tissues.

ROC curves, and the AUC of the expression level of DDIT4 protein (nuclear, cytoplasmic, and membranous) were performed to explore the diagnostic value of the marker in discriminating pancreatic tumor from adjacent normal tissues (Fig. 6). The results of ROC curves demonstrated an AUC of 0.64 (95% CI 0.53–0.75), with 68% sensitivity, and 57% specificity for nuclear expression of DDIT4 in PTs versus adjacent normal tissues. For cytoplasmic expression of DDIT4, an AUC, sensitivity, as well as specificity were 0.70 (95% CI 0.59–0.80), 66%, and 75%, respectively. The membranous expression of DDIT4 also had an AUC, sensitivity, and specificity of 0.62 (95% CI 0.53–0.71), 63%, and 50%, respectively. Table 7 displays the overall diagnostic results for DDIT4 expression.

**Table 7:** Diagnostic evaluation of the DDIT4 in pancreatic tumor patients.

Diagnostic values	Nuclear expression	Cytoplasmic expression	Membranous expression
Sensitivity (95% CI)	68% (61.05%—74.40%)	66% (58.98% -72.53%)	63% (56.42%—70.18%)
Specificity (95% CI)	57% (37.18%—75.54%)	75% (55.13% -89.31%)	50% (30.65%—69.35%)
PLR (95% CI)	1.59 (1.02—2.46)	2.64 (1.38—5.05)	1.27 (0.86—1.87)
NLR (95% CI)	0.56 (0.38—0.82)	0.45 (0.34—0.60)	0.73 (0.48—1.10)
AUC (95% CI)	0.64 (0.53 – 0.75)	0.70 (0.59 – 0.80)	0.62 (0.53 – 0.71)
Cut-off	30	41	26
*P value*	***0.013***	***0.001***	***0.030***

Values in bold and italic are statistically significant.

## Discussion

Pancreatic tumor is recognized as the most fatal and aggressive malignancy among other gastrointestinal cancers, which is estimated to become the second leading cause of cancer-related deaths by 2030^1,46^. PT still has a high death rate, with a 5-year survival rate of around 10%, despite significant advancements and notable improvements in the detection and treatment of many malignancies^7^. By the time of diagnosis, PT often presents at an advanced stage of the disease, and has often metastasized to other organs^47,48^. As a result, it seems sensible to discover innovative biomarkers for early detection, monitoring of recurrence and metastasis precisely, and the reduction of death related to the cancer.

DDIT4 is a main coding RNA whose expressions are altered under the effect of stressor factors, such as endoplasmic reticulum stress, hypoxia, heat shock, and ionizing radiation^20,49^. Research evidence showed that DDIT4 expression was dysregulated in various cancers^50,51^. The multifaceted involvement of DDIT4 in angiogenesis, cancer medication therapy, and the regulation of intricate intercellular signaling networks has led to the proposition that this molecule may possess a dualistic function in the initiation and advancement of cancer^27,35,38,39,52^. Notably, previous studies revealed that DDIT4 has cancer stem cell (CSC) traits, which include self-renewal properties, quiescence, and dysregulated DNA repair^31,38,53–55^. CSCs, which are a subpopulation of cancer cells with self-renewal properties, lead to the maintenance of tumors by generating differentiated tumor cells^56–59^. These features or characteristics of CSCs play crucial roles in heterogeneity, resistance to treatment, recurrence, and metastasis of tumors^56–58^. In addition, the elevated levels of DDIT4 expression increase the survival and growth of tumor cells through stabilizing HIF1α, phosphorylation of P53, and the inhibition of apoptosis pathways, and could also be related to the expression of stem cell markers^27,29,30^.

There was merely one study addressing the clinical significance of DDIT4 in pancreatic tumor, which showed that mutations in the 3'-UTR region of DDIT4 mRNA may affect autophagy by regulating the expression level of DDIT4 in PDAC tissues^49^. Therefore, the expression and function of DDIT4 in PT still need to be clarified. Thus, this is the first study that evaluates the expression, prognostic, and diagnostic value of DDIT4 in both mRNA and protein levels in either PDAC or PNET forms of pancreatic tumor.

Consistent with the result of the bioinformatics analysis, which demonstrated increased mRNA expression of DDIT4, our results in fresh tissue samples showed that mRNA levels of DDIT4 significantly increased in the pancreatic cancer patients when compared to adjacent normal tissues and had a positive correlation with the high grade of the cancer and also with invasion of the lymphovascular. These findings align with the study conducted by Fattahi et al., which showed that the upregulation of DDIT4 in colorectal cancer stem cell-enriched spheroids contributed to tumor advancement and metastasis^31^. Also, upregulation of DDIT4 was observed in GC tissues, which promotes proliferation and tumorigenesis^50^. These data show that increased mRNA expression of DDIT4 is substantially related to more aggressive tumor behavior.

In continuation, our research results indicate a considerable upregulation of DDIT4 protein expression in malignant cells as compared to the adjacent normal samples. Our findings are in agreement with prior studies showing the increased expression of DDIT4 protein in the nucleus, cytoplasm, and membranous tissues in various types of cancer^22,28,31,32,41,50,60,61^. It has also been reported that DDIT4 is expressed mainly in the cell membrane and cytoplasm of normal cells, while it is more expressed in the nucleus of tumor cells^20,62^, suggesting that translocation of the molecule from the membrane into the nucleus may be a risk factor for DDIT4 function changes that lead to oncogenes and may be associated with poor differentiation^22^. Our results confirmed this hypothesis showed that the increased nuclear level of DDIT4 has a positive correlation with invasion to the lymphovascular tissues in PDAC and a high grade of the disease in the PNET samples. Both results revealed that DDIT4 can be considered a risk factor for the progression of pancreatic tumor and plays an oncogenic role in the pancreas. In parallel with the findings, Wei et al. reported that the DDIT4 gene has a p53 transcription-factor binding site, thus it may play a critical role in the p53-dependent tumorigenesis in the nucleus^63^. Similarly, other studies have stated this fact that the high nuclear expression of DDIT4 protein is associated with advanced histological grade and enhances cancer proliferation and tumorigenesis^32,33,64^. To confirm the results, a review article by Ding, F., and colleagues collected the recent information regarding the roles played by DDIT4 in the progression of tumors and proved that the nucleus expression of DDIT4 is associated with tumor deterioration^42^. Therefore, DDIT4's functionality may be dependent on its precise subcellular location inside pancreatic cells. Notably, higher levels of DDIT4 nuclear expression have been associated with more aggressive tumor behavior.

Moreover, although there were no significant differences between the expression of DDIT4 and survival in either PDAC or PNET patients, the pancreatic cancer patients with the high nuclear expression of DDIT4 had shorter survival duration compared to those with the low nuclear expression of DDIT4, which was consistent with our clinicopathological findings. Our findings are parallel to the previous studies, concluding that DDIT4 expression is not a prognostic factor for tumors^32,50^. Besides, our survival analysis demonstrated that the patients with high cytoplasmic and membranous expression of DDIT4 had a higher survival rate in PDAC cases. Besides, our survival analysis showed that the patients with high cytoplasmic and membranous expression of DDIT4 had a higher survival rate in PDAC cases. However, there was no difference in DSS or PFS follow-up months between PNET patients with high cytoplasmic or membranous expression of DDIT4 and low cytoplasmic or membranous expression. While cytoplasmic overexpression of DDIT4 protein was shown in several studies to be a poor prognosis factor for tumor progression in ovarian carcinoma, acute myeloid leukemia, OSCC, and BUC^22,28,33,65^. Moreover, in this study, distant metastasis was indicated as an independent prognostic factor for OS, DSS, and RFS in PDAC patients. Previous studies reported that metastasis has been significantly associated with disease progression as well as unfavorable outcomes^66,67^. Distant metastasis has been considered as the major cause of the high death rate in patients with PT^68^.

As well, in terms of diagnosis, it has been shown that less than twenty percent of pancreatic cancer patients can be surgically removed at the time of their initial diagnosis^69^. Currently, carbohydrate antigen 19–9 (CA19-19), which is extensively used in clinical practice, has been authorized by the US Food and Drug Administration (FDA) as the only biomarker for the detection of pancreatic tumor. Nevertheless, the specificity of the current biomarker is limited, thus necessitating the urgent exploration and creation of new biomarkers^70^. Hence, we aimed to assess the diagnostic accuracy of DDIT4 and its sensitivity and specificity in discriminating PT from adjacent normal tissue samples. Our results revealed that the expression levels of DDIT4 protein proved that the molecule may be considered the potential marker for diagnosis of pancreatic cancer patients with high sensitivity. Of note, these data support the direct role of DDIT4 in pancreatic tumors, which can be used to diagnose the PT. However, we suggest assessing the concentration of DDIT4 in the serum of PT patients, a noninvasive detection method, as a potential diagnostic index, which might be an innovative approach.

Our study presents persuasive and consistent evidence regarding the clinical significance of DDIT4 in PT patients. However, we must recognize some limitations. These include the relatively small number of PDAC and PNET samples utilized in the study, as well as the short duration of follow-up. These limitations may cast doubt on the prognostic role of DDIT4. Therefore, it is crucial to evaluate the expression of DDIT4 in a larger sample size and extend the duration of follow-up to confirm the diagnostic and prognostic role of this marker.

Furthermore, we also acknowledge that we did not explore the detection mechanism and signaling pathways of DDIT4 in PT. To gain better insight into the mechanisms and functions of DDIT4 in PT patients, it is advisable to determine the signaling pathways involved.

## Conclusion

In summary, bioinformatics analysis confirmed that DDIT4 in mRNA level has increased in PT and can be considered as a potential prognostic marker. Our findings showed that DDIT4 at the level of mRNA and protein was upregulated in the PT compared to the adjacent normal tissues. In contrast to its cytoplasmic and membranous expression, higher nuclear level of DDIT4 is associated with more aggressive behavior, advanced disease, and a worse survival rate. Therefore, the nuclear expression of DDIT4 could serve as a promising marker and a potential therapeutic target for PT patients. However, further investigations should be conducted regarding the molecular mechanisms of DDIT4 in the progression of pancreatic tumor.

## Materials and methods

### Data mining through bioinformatics tools (Investigation of DDIT4 in patients with pancreatic adenocarcinoma)

Gene Expression Profiling Interactive Analysis (GEPIA2) is an online bioinformatics tool for expression data from RNA sequence for The Cancer Genome Atlas (TCGA) and the Genotype-Tissue Expression (GTEx), tumor and normal samples^71^. In the primary search for the investigation of DDIT4 expression in sample tissues from patients with pancreatic adenocarcinoma, GEPIA2 was performed. Moreover, GEPIA2 was applied to evaluate the prognostic potential with the overall survival curve of the DDIT4 expression in these samples which was stratified according to the median expression of DDIT4 in GEPIA2.

### Patient’s characteristics and sample collection

In this cross-sectional study, 27 PDAC fresh tumor tissues and their adjacent normal tissue samples from 2020 to 2021 were collected from Firoozgar Hospital, a university-based hospital in Tehran, Iran. Patients who had undergone surgery without any prior chemotherapy/radiotherapy were enrolled in this study. The samples were transferred to the laboratory, and frozen in liquid nitrogen. Patient information, including gender, age, TNM stage, histological grade, lymphovascular invasion, perineural invasion, and lymph node metastasis were recorded.

Moreover, 200 formalin-fixed paraffin-embedded (FFPE) tissue samples from PT patients were collected from two university-based referral hospitals (Firoozgar and Imam Khomeini) in Tehran, Iran, between 2010 and 2021. These samples comprised two primary classifications of PT (PDAC and PNET) that we encounter within our hospitals. None of the surgical participants received adjuvant therapy. Hematoxylin and Eosin (H & E) stained slides and the medical archival documents were acquired to extract clinical and pathological parameters, comprising age, gender, tumor size (maximum tumor diameter), histological grade, TNM stage, distant metastasis, tumor recurrence, margin involvement, macroscopic tumor extension, lymphovascular invasion, perineural invasion, and lymph node metastasis. However, it is important to note that chemotherapy was virtually always given following surgery to all of our patients in accordance with recommendations, thus we did not include it as a variable in our analysis. Furthermore, 27 adjacent normal tissues were utilized to determine the expression of DDIT4 compared to cancerous samples. OS represents the duration starting from the surgery date until the date of either death or the last follow-up visit. DSS calculated from the date of surgery to the date of death related to the patient’s tumor and PFS defined as the date of the primary surgery and the last follow-up visit for the patient with no evidence of disease, metastasis or recurrence, was also collected. Tumor staging was performed according to the pTNM classification for pancreatic tumor^72^. This study received its ethical approval (Code: IR.IUMS.REC.1400.350.) from the Research Ethics Committee of Iran University of Medical Sciences. All procedures, including obtaining informed consent from each human participant before surgery, were by the above-mentioned ethical standards. Thus, we confirm that all research was performed by relevant guidelines and regulations.

### RNA extraction and cDNA synthesis

Total RNA was extracted from fresh tumor tissue samples, as well as normal adjacent tissues, using RNA Minipreps Super Kit (Bio Basic, Canada) according to the manufacturer’s instructions. All extracted RNAs were measured in quantity and quality by Nanodrop (ThermoFisher Scientific, USA). Then RNA was reverse transcript into cDNA using the cDNA Synthesis Kit (Yekta Tajhiz Azma, YT4500, Iran).

### Real-time- quantitative polymerase chain reaction (RT‐qPCR)

The specific primers (DDIT4, TPTEP1) for amplification with real-time- quantitative PCR (RT-qPCR) were designed as described previously^31^. RT-qPCR was performed using the Rotor-Gene Q Light Cycler (Qiagen, Germany) by the SYBR Green qPCR master mix (Yekta Tajhiz Azma, YT2551, Iran). After that, the mRNA level of the reference gene, GAPDH, was used as an internal control in each sample to normalize the relative expression levels of mRNAs. RT-qPCR data were analyzed using the comparative Ct method (known as the 2^-ΔΔCt^ method) to present relative expression levels of the genes^73^. The sequence-specific primers are shown in Table 8.

**Table 8:** Primer sequence of genes for RT-qPCR.

Gene of interest	Gene name	Primer Sequence (5´ → 3´)
Target genes	DDIT4	F: CTTTGGGACCGCTTCTCGTC
		R: GGTAAGCCGTGTCTTCCTCCG
	TPTEP1	F: AGCCGCAGACAAAAGACCTCGG
		R: CCACCAAACAGGCTTCGTGTGA
Housekeeping gene	GAPDH	F: GCACCGTCAAGGCTGAGAAC
		R: TGGTGAAGACGCCAGTGGA

### Tissue microarray (TMA) construction

Pancreatic tissue TMA blocks were constructed as described previously^74,75^. In brief, two experienced pathologists (B.B. and P.B) marked the three most representative tumor areas in different parts of each block after matching them with corresponding H&E slides. The chosen tumor sections of each block were then punched out with a 0.6 mm diameter and placed into a fresh recipient paraffin block using a precision arraying tool (Tissue Arrayer Minicore; ALPHELYS, Plaisir, France). Next, completed TMA blocks were cut at 4-μm sections and transferred to adhesive slides. To avoid tumor heterogeneity as a significant concern during the TMA method and increase the accuracy and validity of the data analysis, TMA blocks were constructed in three copies from each tumor specimen, and the mean scores of the three cores were calculated as the final score^76–78^. Notably, to compare the expression patterns of DDIT4 with tumor tissue specimens, adjacent normal tissue samples were also included in each TMA block^79^.

### Immunohistochemistry (IHC) staining

DDIT4 expression was evaluated using the IHC method as previously described^80,81^. First, all TMA sections were deparaffinized at 60 °C for 40 min and then rehydrated with xylene and graded ethylic alcohol. To block endogenous peroxides and non-reactive staining, slides were treated with 3% H2O2 for 20 min at room temperature. After washing the slides in Tris Buffered Saline (TBS), antigens were retrieved by immersing the tissue slides in citrate buffer (pH 6.0) for 10 min in an autoclave. Next, the slides were washed in TBS three times again and were stained by the primary antibody of anti-DDIT4 (Biorbyt, Cambridge, UK) with a dilution of 1:80 overnight at 4 °C. Besides, rabbit immunoglobulin IgG (Invitrogen, Thermo Fisher Scientific, Waltham, MA, USA) was utilized at the same dilution of primary antibody for the isotype control test. The next day, following three times washing in TBS, TMA sections were treated with secondary antibody, including EnVision _TM_Mouse/Rabbit PolyVue HRP reagent (Dako; code K5007; Denmark), for 30 min at room temperature. Slides were incubated by 3,3′-diaminobenzidine (DAB) (Dako, Glostrup, Denmark) as a chromogen and, after washing in TBS, counterstained by Mayer’s hematoxylin dye (Dako, Glostrup, Denmark) for 15 min to visualize the antigen. Eventually, the slides were dehydrated the same in graded ethylic alcohol, cleared in xylene, and mounted for examination. Furthermore, TBS was utilized instead of the primary antibody as a negative control and normal liver tissue as a positive control.

### Evaluation of immunostaining and scoring score

The staining of DDIT4 protein marker on TMA slides was scored by two independent pathologists (B.B and P.B.) using a semiquantitative scoring system, blinded to the clinicopathological and survival parameters of the patients. To evaluate the expression of DDIT4, three scoring systems were used as follows: staining intensity, the percentage of positive tumor cells, and the H-score. The intensity of staining was scored based on a 4-point scale (negative or non-staining = 0, weak = 1, moderate = 2, and strong = 3), and the percentage of positive tumor cells was assessed and categorized into four groups (< 25, 25–50%, 51–75%, and > 75%). Then, the overall score was attained through the Histochemical score (H-score) by multiplying the staining intensity and the percentage of the positive cells, which ranged from 0 to 300 in each case. The H-scores were classified into two groups based on median: low expression (≤ median) and high expression (> median).

### Statistical analysis

All data were analyzed using "statistical software SPSS" version 25.0 (SPSS, Inc., IBM Corp, USA). The categorical and the quantitative data were represented by N (%) and mean (SD) or median (Q1, Q3), respectively. Pearson's chi-square and Spearman's correlation tests were carried out to determine the significance of association and correlation between DDIT4 protein expression and clinicopathological characteristics. Kruskal–Wallis and Mann–Whitney U tests were conducted to compare the study groups pairwise. Survival curves were plotted by applying the Kaplan–Meier method with a 95% confidence interval (CI) and compared survival outcomes between low and high marker expression by the log-rank test. Furthermore, the univariate Cox proportional hazards regression model was utilized to determine which variables impacted DSS or PFS. Then, those parameters that significantly affected the survival in univariate analysis were enrolled in multivariable Cox proportional hazards regression analyses. To evaluate the diagnostic value of the DDIT4 protein, the area under the ROC curve (AUC), sensitivity, specificity, positive likelihood ratios (PLRs), and negative likelihood ratios (NLRs) were calculated by using receiver operating characteristic (ROC) curve analysis. In all parts of the analysis, *P* < *0.05* was considered statistically significant difference.

GraphPad Prism version 8.4.3 software (GraphPad Software, La Jolla, CA, USA) was used for making the boxplots and scatterplots.

### Ethical approval

All procedures performed in this study were in line with the ethical standards of the institution at which this study was conducted. The Research Ethics Committee of Iran University of Medical Sciences issued IR.IUMS.REC.1400.350 for this study.

### Informed consent

Informed consent was obtained from all individual participants, parents or legally authorized representatives of participants under legal age years old at the time of sample collection with routine consent forms.

## Data Availability

The analyzed data during the current study are available from the corresponding author on reasonable request.
